# Human monkeypox infection threat: A comprehensive overview

**DOI:** 10.1371/journal.pntd.0011246

**Published:** 2023-04-20

**Authors:** Yue Kang, Yue Yu, Silu Xu

**Affiliations:** 1 College of Pharmacy, Nanjing University of Chinese Medicine, Nanjing, Jiangsu, China; 2 School of Pharmacy, Fujian Medical University, Fuzhou, Fujian China; 3 Department of Pharmacy, Jiangsu Cancer Hospital & Jiangsu Institute of Cancer Research & The Affiliated Cancer Hospital of Nanjing Medical University, Nanjing, Jiangsu, China; Ben-Gurion University of the Negev, ISRAEL

## Abstract

**Background:**

In addition to the COVID-19 waves, the globe is recently facing global monkeypox (MPX) outbreak. As the daily confirmed cases of MPX infection across epidemic and nonepidemic countries are increasing, taking measures to control global pandemic remains crucial. Therefore, this review aimed to provide fundamental knowledge for the prevention and control of future outbreaks of this emerging epidemic.

**Methods:**

The review was conducted using PubMed and Google Scholar databases; the search terms used were “monkeypox,” “MPX tropism,” “replication signaling of MPX,” “biology and pathogenicity of MPX,” “diagnosis of MPX,” “treatment of MPX,” “prevention of MPX,” etc. The update epidemic data were collected from the websites of the World Health Organization (WHO), United States Centers for Disease Control and Prevention (CDC), and Africa Center for Disease Control and Prevention (ADCC). High-quality research results published in authoritative journals were summarized and preferred cited. Excluding all duplicates, non-English published references, and irrelevant literature, totally 1,436 articles were assessed for eligibility.

**Results:**

It is still difficult to diagnose the patient as MPX simply based on clinical manifestations; therefore, under this situation, employing polymerase chain reaction (PCR) technology to provide confirmed evidence for the diagnosis of MPX seems to be the preferred and indispensable strategy. The treatment approach for MPX infection is mainly symptomatic and supportive; anti-smallpox virus drugs including tecovirimat, cidofovir, and brincidofovir can be employed in severe cases. Timely identification and isolation of confirmed cases, cutting off dissemination routes, and vaccination of close contacts are effective measures to control MPX. Also, smallpox vaccines (JYNNEOS, LC16m8, and ACAM2000) can be under consideration due to their immunological cross-protection among Orthopoxvirus. Nevertheless, given the low quality and scarcity of relevant evidence of current antiviral drugs and vaccines, deeply seeking for the MAPK/ERK, PAK-1, PI3K/Akt signaling, and other pathways involved in MPX invasion may provide potential targets for the treatment, prevention, and control of the epidemic.

**Conclusions:**

In response to the current MPX epidemic, the development of vaccines and antiviral drugs against MPX, as well as the rapid and precise diagnostic methods are still urgently needed. Sound monitoring and detection systems should be established to limit the rapid spread of MPX worldwide.

## Author summary

The daily increasing reported cases of MPX infection have set off the alarm for a global pandemic. In this review, we report the history of MPX infection and the biology and pathogenicity of MPX. The report chronicles the viral tropism and the role of signaling in MPX infection. We also summarize the current diagnosis approaches, and the therapeutic options available for the treatment and management, all of which will bridge the knowledge gap on MPX infection, provide a theoretical basis for combating the outbreak.

## Introduction

Monkeypox (MPX) is a zoonotic disease caused by the MPX virus, originating from monkeys in the rain forests of Central and Western Africa [1]. MPX exists in 2 main types of genetically evolved strains—the Central African strain and the West African strain; recently, the third strain was derived from the West African clade. MPX was first isolated from monkeys in a laboratory in 1958; this case was of a 9-month-old baby who was admitted to Congo, with the suspicion of smallpox. Most of the MPX-infected cases have since then occurred in the Congo, Central Africa, and West Africa [2]. Since 2017, an outbreak of MPX has occurred in Nigeria, with more than 700 cases. The first outbreak of MPX outside of Africa occurred in the United States of America (USA) in 2003. From 2018 and 2022, increasing numbers of cases of MPX were recorded among tourists traveling from Nigeria to other countries. To date, no clinically proven specific treatment has been established for MPX, which makes it urgent to develop effective measures for treating, controlling, and preventing [3].

Understanding the etiology and pathogenicity of MPX, the interactions of MPX with host cells is a prerequisite for the management of antiviral measures. Considering the severity of MPX outbreaks all across the world, we screened and summarized the current literature reporting the MPX tropism, the replication cycle of MPX, and the signaling involved in the invasion, as well as the diagnosis, treatment, and prevention of MPX, with the hope of establishing measures toward better controlling this disease.

## Methods

### MPX literature research

**Fig 1:**
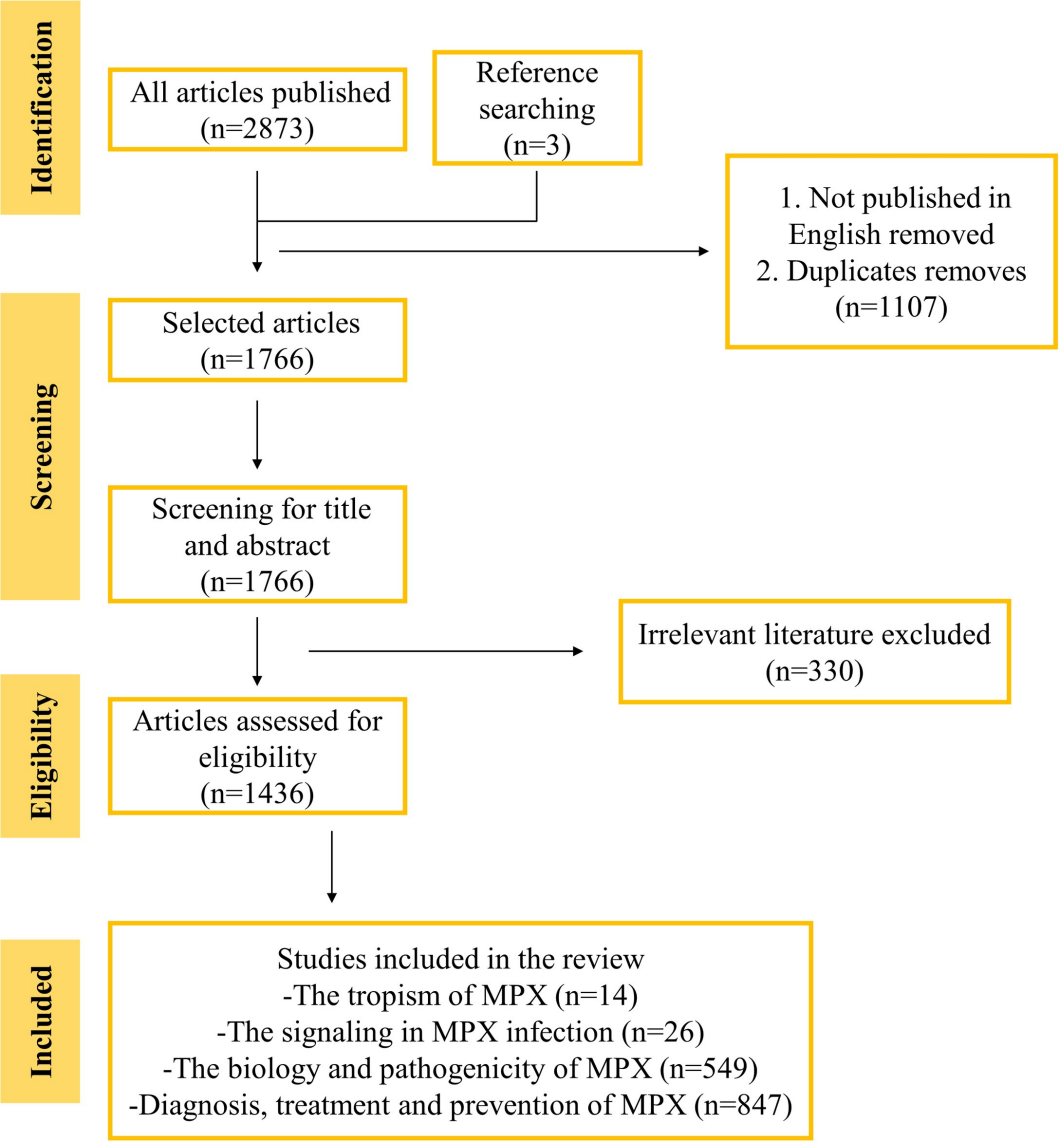
The study selection.

Literature was searched in the PubMed and Google Scholar, using the following search terms: MPX, MPX tropism, MPX replication signaling, biology and pathogenicity of MPX, etc. Check the references from all sources to find eligible articles. The reference lists of all articles published in peer reviewed journals selected were reviewed, 94 not published in English were excluded, all the duplicates were excluded, and totally 1,766 references were identified. Among these eligible references, only 14 summarized or investigated the tropism of MPX, 26 chronicled the signaling in MPX infection and identified potential host targets of therapeutic interest, 549 records regarding the biology and pathogenicity of MPX, and 847 summarized the diagnosis, treatment, and prevention of MPX (**Fig 1**). High-quality research results published in authoritative journals were summarized and preferred cited in the review.

In addition to search sources in the PubMed and Google Scholar, the latest outbreak progress was monitored from the websites of the World Health Organization (WHO), United States Centers for Disease Control and Prevention (CDC), and Africa Center for Disease Control and Prevention (ADCC); we update our charts according to new cases added daily.

### Data summary

The information regarding the MPX-infected animals and the host range of MPX from the selected literatures was summarized in the tables. Due to the incompleteness of MPX research field, we concluded current signaling pathways involved in the poxvirus infection to provide guidance for the treatment of MPX. In addition, we summarized the diagnostic methods, treatment, and prevention of MPX and application for various settings in the tables.

## Results

### The epidemiology investigation

MPX is a member of *Orthopoxvirus* (OPV) genus. OPV family is divided into *Chordopoxvirinae* and *Entomopoxvirinae* families; they are the largest DNA viruses that have ever been reported. MPX is surrounded by a lipoprotein envelope, containing a linear double-stranded DNA genome in the biconcave-like core; the external surface is ridged in parallel rows, sometimes arranged helically. Viral particles are usually encapsulated (outer membrane viral particles EEV). The intracellular mature virus particle (IMV) form of the virus contains a different envelope and is infectious. The whole viral replication cycle occurs in the cytoplasm; all proteins required for viral DNA replication, transcription, virion assembly, and expulsion are encoded by the viral genome [4].

**Fig 2:**
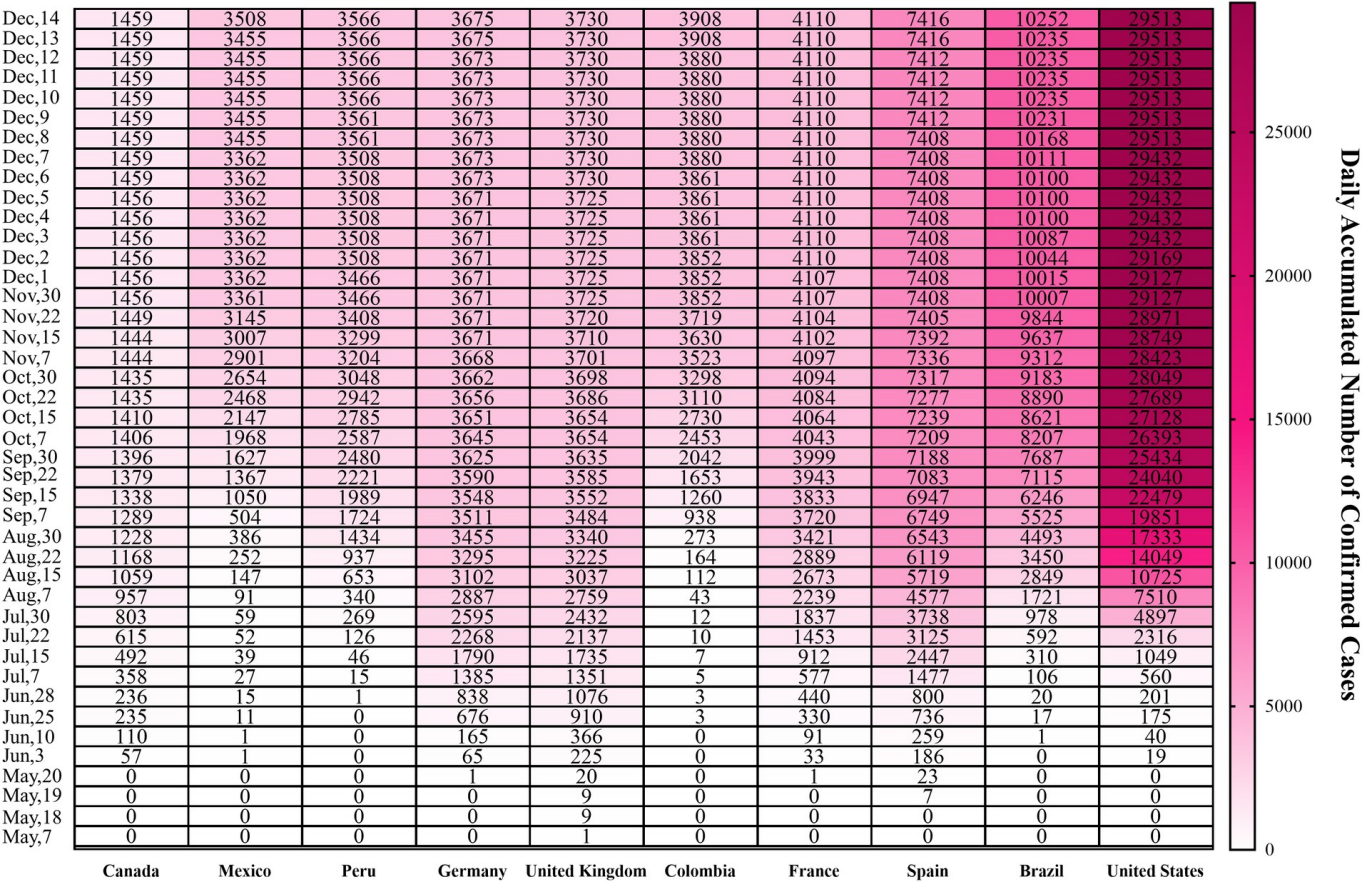
Cumulative confirmed cases, by date of confirmation in countries with total confirmed cases ranking top 10 till December 14, 2022.

The MPX epidemic sweeping Europe in 2022 began on May 6; a rare case of MPX infection has been confirmed in England, following the announcement of a confirmed case by British Health and Safety Authority. Europe and the US and other countries have also reported confirmed or suspected cases. Massachusetts reported a case of MPX; the patient was an adult male who had recently traveled to Canada; it was the first confirmed case in the US in 2022. Subsequently, Canada reported 2 confirmed cases, and Australia confirmed the first MPX case [5]. According to the latest data provided from WHO, as of December 14, countries with the cumulative confirmed cases ranking top 10 are US (29,513), Brazil (10,252), Spain (7,416), France (4,110), Colombia (3,908), United Kingdom (3,730), Germany (3,675), Peru (3,566), Mexico (3,508), and Canada (1,459) (**Fig 2**), indicating that there is an urgent need to take measures to prevent and control the epidemic.

### Primary literature review on the OPV tropism

Under the current epidemic condition, a host that can be infected with MPX should be categorized to facilitate the prevention and control of the epidemic. Accordingly, we found that 14 references discussed on the tropism of OPV and identified 3 levels of viral tropism: cellular tropism, host or organism tropism, and tissue tropism [6,7]. Each of these levels of tropism determines whether the virus will exhibit tropism toward the host species. Generally, the virus is relatively less pathogenic in the host and can only cause a subclinical level of infection. However, zoonotic infections are usually highlighted only after species transfer, which increases viral pathogenicity or generates novel diseases. Tissue and host tropism majorly influences the ability of a virus to spread among hosts. These 3 levels of tropism are highly interdependent in terms of determining whether it possesses the ability to infect the host. There exists a wide range of genera, species, families, and orders of mammals affected by MPX [8,9], suggesting a wide host tropism of MPX (**Table 1**). Meanwhile, high levels of viral DNA were monitored in the eyes, oral and nasal cavities of MPX-infected animals, suggesting that MPX has a wide tissue tropism. The members of the genus OPV have different host tropism profiles; this variability may enhance the host–virus interactions, reduce pathogenicity, and distinguish the immune evasion molecules.

**Table 1:** The reported MPX-infected animals.

Reported animals infected with MPXV	Countries	Diagnostic methods	Viral strain
Sooty mangabey monkey	Côte d’Ivoire	PCR assay	West African strain
Gambian-pouched rat	Africa	PCR assay	West African and Central African stain
Rhesus macaques	Copenhagen	Serological test	West African strain
Sun squirrel	Zaire	Antibody detection test	Central African stain
Elephant shrew	DR Congo	Serological test	Central African stain
African hedgehogs	Africa	PCR assay, antibody detection test	West African Strain
African dormice	USA	PCR assay	Central African stain

The binding specificity of viruses depends on their specific receptors distributed across the host cell surface. Vaccinia virus (VACV), a prototypical member of *Poxviridae*, reportedly utilizes glycosaminoglycans, the extracellular matrix protein laminin, and/or integrin β1 on the host cell membrane [10]. Chemokine receptors were reported to be used by poxviruses for infecting several intracellular pathogens, especially migratory leukocytes [11]. Nevertheless, no virus tropism specificity has been reported in poxviruses. In fact, they can bind to and enter a variety of mammalian cells to complete the replication cycle. OPV can bind and penetrate both permissive and restrictive cells, although the downstream intracellular events are easily suspended in restrictive cells [12]. Therefore, poxviruses may invade a wide range of mammalian cells, but the ability of different poxviruses to complete the entire replication cycle varies significantly across cells originating from different lineages or species (**Table 2**).

**Table 2:** The host range of poxviruses.

Poxvirus	Genus	Host	Zoonic host
Smallpox	Orthopoxvirus	Human	None
Molluscum contagiosum	Molluscipoxvirus	Human	None
Monkeypox	Orthopoxvirus	Rodents	Human and monkey
Cowpox	Orthopoxvirus	Rodents	Humans, cows, cats, most mammalian cells
Vaccinia	Orthopoxvirus	Unknown	Wide range
Ectromelia	Orthopoxvirus	Rodents	Mice
Tanapox	Yatapoxvirus	Rodents	Human and monkey
Myxoma	Leporipoxvirus	Rabbit	Rabbit

### Poxvirus-induced signaling pathways

**Fig 3:**
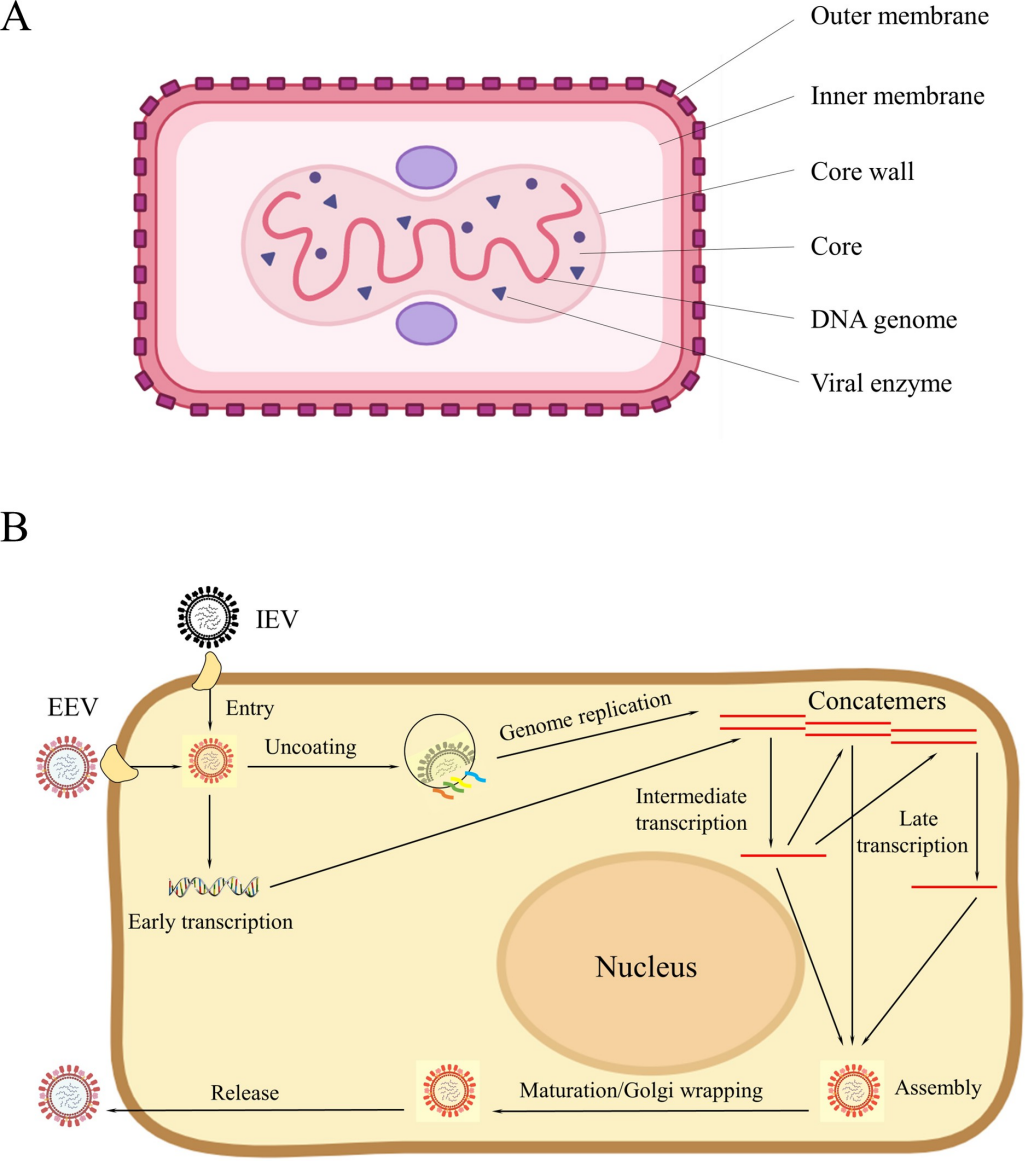
(**A**) Graphic representation of poxvirus. (**B**) The replication cycle of poxviruses. All the events including attachment, entry, uncoating, genome replication, intermediate transcription, late transcription, assembly, maturation/Golgi wrapping, and release of virus are performed in the diagram. EEV, extracellular enveloped virus; IEV, intracellular enveloped mature virus.

The induction or manipulation of host cell signaling cascades is critical for successful poxvirus infection as it influences viral pathogenesis. Unlike influenza virus, relying on the low pH-dependent endosome attachment system, the pH-nondependent attachment system is more important during poxvirus replication (**Fig 3**). The OPV invasion causes the activation of downstream signaling molecules, including MAPK/ERKs, JAKs, STATs, rac/rho, etc. [13,14]. For instance, VACV causes host cells to secrete a peptide called VACV-inducible growth factor (VGF), and these cells can compete for binding to the epidermal growth factor receptor (EGFR) on the cell surface. VACV can utilize the mitogenic-signaling potential of EGFR to activate the MAPK–ERK cascade. The activation of the MAPK/ERK induces a key enzyme—thymidine kinase (TK) for viral replication; the viral yield was significantly reduced when the TK inhibitors were employed, thereby underscoring the involvement of MAPK/ERK signaling in the poxvirus replication cycle, and making targeting the MAPK/ERK cascade promising for the development of anti-poxvirus options [15]. PD98059 and U0126, which are commonly used for inhibiting the MAPK/ERK cascade, was reported to successfully prevent VACV infection [16]. In addition, the second-generation MAPK/ERK inhibitors were recently under Phase II of clinical trials [17], suggesting that optimized options may become available or known in the near future.

PAK-1 is a member of STKs family that serves as downstream effector of small GTP-binding proteins to respond to the motility and cytoskeletal rearrangements, the activation of transcription factors, and the pro- and anti-apoptotic processes [18]. MPX and VACV share a dependency on the activity of PAK-1/Raf1 for promoting the replication progress [19]. Considering the role of PAK-1 in the life cycle of several pathogenic viruses, PAK-1 inhibitors have been considered as promising antiviral agents. However, targeting PAK-1 may inadvertently promote virus growth; therefore, a better understanding of these processes is essential to determine whether PAK-1 inhibition can avoid these problems before considering PAK-1 inhibitors as antiviral drugs.

The integrin family is the predominant molecule mediating the adhesion of stem cells to the extracellular matrix and regulating signal transduction to maintain important cellular functions [13]. Integrin β1 regulates different intracellular kinase activation pathways through interaction with the extracellular matrix, which is crucial for the VACV entry into HeLa. VACV binds to integrin β1, activates the downstream PI3K/Akt kinase pathway, and induces viral endocytosis. Blocking PI3K reduces the viral yield in an integrin β1-dependent manner [20]. Therefore, the development of antiviral agents that target PI3K/Akt is encouraging [21].

Cyclic GMP-AMP synthase (cGAS) is the key cytosolic DNA sensor that triggers interferon gene-stimulating factor (STING) and produces type-I interferon (IFN). Ectromelia virus (ECTV), the ideal model for OPV infection analysis, was reported to trigger the production of a type-I IFN response in L929 and RAW264.7 cells [22]. Mice lacking cGAS or STING had a lower level of type-I IFN and a higher viral load, indicating the involvement of the cGAS-STING signaling pathway in type-I IFN production and ECTV replication [23]. The present results can provide insights into the optimization of poxvirus-based vaccines for their clinical application against infectious diseases and cancer.

### Diagnostic methods

#### Laboratory diagnosis

MPX is transmitted from person to person, which makes its early and rapid diagnosis critical to control the disease outbreak. The clinical manifestations of MPX are difficult to distinguish from those of other poxvirus-induced diseases. Thus, a combination of multiple methods focused on “clinical symptoms, epidemiology, and laboratory tests” is necessary for an accurate diagnosis. Of these, laboratory testing techniques are essential to confirm the diagnosis of MPX. **Table 3** lists the commonly performed assays for MPX diagnosis, which includes virus isolation and identification, nucleic acid detection, and immunological detection.

Polymerase chain reaction (PCR) is the preferred laboratory test in this situation, considering its accuracy and sensitivity. However, the combination of multiple testing methods can facilitate an increased detection rate of positive viruses and obtain more information about the virus, so as to provide evidence for the determination of the epidemiology, the source of infection, and the mode of transmission of the virus.

**Table 3:** Laboratory diagnostic methods of MPX.

Tests	Virus isolation and identification		Nucleic acid testing			Immunological testing
	Viral Culture	Electron microscopy	Real-time PCR	Whole-genome sequencing	LAMP	ELISA
Description	The virus is grown and isolated from a patient sample	Used to morphologically identify pox viruses	The detection of MPX DNA	Obtaining the proto-nucleic acid sequence of MPV directly from the samples	A new, thermostatic nucleic acid amplification technique without thermal cycling	Detection of the presence of antibodies to orthopoxvirus
Sample used	Rash, herpes, scabs, oropharyngeal or nasopharyngeal secretions	Suspension made of herpes fluid, pustules or scabs	Skin lesions–the roof or fluid from vesicles and pustules, and dry crusts			Serum specimen
Pros	·High accuracy ·Important for monitoring new trends in epidemics, studying new viruses and vaccine development	·OPV can be confirmed by morphological observation of the virus ·Direct observation without specific biological reagents ·Can be used as a complement to other assays to elucidate the life cycle of viruses and contribute to the development of antiviral drugs and vaccines	·Has high sensitivity and good specificity for real-time detection of amplification products due to the addition of specific fluorescent-labeled probes, which can effectively identify MPV branches or distinguish other OPV ·The gold standard for confirming a positive diagnosis of MPX, allowing direct testing of patient specimens for rapid, early diagnosis	·Helping better understand the epidemiology, source of infection, and mode of transmission of MPX by detecting the DNA sequence ·Sequencing of up to several million DNA molecules at a time ·No need to isolate and culture pathogens and does not rely on known nucleic acid sequences, allowing direct sequencing of specimens for identification ·High throughput, low detection limit, high accuracy, and can obtain abundant information	·Can realize rapid screening of MPX, effectively diagnose MPX and distinguish OPX types, and can distinguish Congo basin strains and West African strains ·Has the characteristics of high efficiency, simplicity, high specificity, and no need for thermal cycling equipment ·Detection cost is far lower than RT-PCR	·Suitable for large-scale seroepidemiological screening of high-risk populations and as a cotest with RT-PCR for diagnostic of MPX ·Can be used to assess previous exposure to orthopox virus, including pathogenic or smallpox vaccination ·Easy to operate and has better protection for medical staff
Cons	·Long test cycle ·High demand for experimental techniques and conditions, the operations need to be performed by experienced technicians	·Expensive, low sensitivity, long cycle time, and the operation is complex, must be equipped with professional technicians to use in the electron microscope laboratory	·Can be affected by the quality of specimen collection, storage, or nucleic acid extraction, and false negative results may occur ·Concerns about sample contamination, and demand for high-cost equipment and reagents	·Costly, high demand for downstream processing for sequencing data, is not suitable for large-scale detection ·The accuracy mainly depends on the scope and integrity of the database	·Having the important step of designing multiple pairs of primers and enzymes, which requires a lot of optimization and verification to find specific primers and enzymes ·DNA polymerase is sensitive to temperature changes and prone to false positive results ·Easy to form aerosols that cannot be easily eliminated in the laboratory environment	·Low specificity since MPX has antigenic cross-reactivity with other pox viruses, and the results are susceptible to previous smallpox vaccination and may give false positive results ·Cannot achieve early diagnosis, can only be applied to late auxiliary diagnosis
Applications	Virus research, development of vaccines and antiviral drugs		The preferred method for laboratory diagnosis of MPX	The gold standard for distinguishing MPX from other orthopoxvirus, identify the MPX strain	MPX rapid screening, applicable to inspection and quarantine ports and grassroots medical institutions	Seroepidemiological screening of large high-risk populations or late ancillary diagnosis

ELISA, enzyme-linked immunosorbent assay; LAMP, loop-mediated isothermal amplification; MPX, monkeypox; OPV, Orthopoxvirus; RT-PCR, real-time PCR.

#### Viral isolation and identification

MPX isolation culture is the process of culturing and characterizing the virus through cell inoculation and chicken embryo inoculation after appropriate processing of the sample suspected of carrying the virus. The isolated and cultured MPX can be employed for the preliminary determination of the virus species and its virulence and specificity by electron microscopy, etch formation test, and enzyme-linked immunosorbent assay (ELISA).

Detection of MPX by electron microscopy is the preferred method to understand the viral morphology in detail. In this method, herpes fluid, pustules, or scabs were collected to prepare the viral suspensions, and the morphological characteristics of the virus particles were observed by electron microscopy. MPX and other OPV were found to be similar in size and shape, either brick-shaped or oval, measuring 200 × 250 nm in size, and presenting with a vesicle membrane [2].

#### Nucleic acid testing

Nucleic acid testing involves the detection of MPX nucleic acids in specimens obtained from skin lesions by using nucleic acid amplification assays. A variety of nucleic acid assays are available.

Real-time (RT) PCR is the gold standard for confirming a positive diagnosis of MPX. A positive diagnosis is confirmed by the detection of a MPX genomic fragment from the skin lesion specimens of MPX patients. Typically, the gene sequences from the conserved regions of the virus are selected as targets for amplification; for example, the extracellular vesicle protein gene *B6R* [24], the DNA polymerase gene *E9L* [25], the DNA-dependent RNA polymerase subunit 18 (*RPO18*) gene [26], and the complement-binding proteins C3L [27], F3L [26], and N3R [28].

Whole-genome sequencing is the gold standard for distinguishing MPX from other OPVs [29,30]. By sequencing the entire genome of the isolated MPX, the genus and species of this virus and the genetic evolutionary branching strains could be accurately determined and the related mutations could be identified [2]. Sequencing of MPX is recommended for positive specimens.

In addition, restriction length fragment polymorphism (RFLP) [31], loop-mediated isothermal amplification (LAMP) [32], and recombinase polymerase amplification (RPA) [33] are all established methods for MPX nucleic acid detection.

#### Immunological testing

When clinical manifestations and epidemiology indicate MPX infection and the PCR results are negative, immunological testing may facilitate the identification of past infections. ELISA is the preferred method for serum antibody detection through antigen–antibody specific binding reactions to analyze the immune reaction, and it can be used when there is no virological sample. The specific IgM and IgG can be determined after 5 or more than 8 days of the appearance of the rash in infected patients [2]. A confirmed diagnosis requires a double serum specimen in the acute and recovery phases, with a 4-fold or higher increase in the IgG antibody titer during the recovery phase when compared to that in the acute phase, and the specificity of ELISA is low due to the immune cross-reactivity between MPX and other OPV [2,34]. In addition, western blot [35] and immunohistochemistry [36] can be employed for MPX detection, but not in routine analysis.

#### Clinical diagnosis

The initial clinical diagnosis is based on a temperature of ≥38.5°C, with fever and swollen lymph nodes acting as typical clinical symptoms in patients with MPX. Within 1 to 10 days, skin rashes occur almost simultaneously on the face, palms, and feet, and the number of lesions ranges from a few to several thousand; the oral mucosa, the genitalia, and the conjunctiva, as well as the cornea, are all affected [2,37,38]. Clinically diagnosing MPX based on clinical symptoms is significant for identifying the suspected cases. In a study that did not conduct laboratory confirmation, 645 clinical cases were identified, exhibiting a high sensitivity (93% to 98%) but low specificity (9% to 26%) rate [39].

On May 22, 2022, WHO established diagnostic criteria for MPX in non-MPX endemic countries in patients of any age presenting with an acute rash of unknown origin or single/multiple skin lesions with one or more of the following signs or symptoms: acute fever (>38.5°C), headache, lymphadenopathy (enlarged lymph nodes), myalgia (muscle and body pain), back pain, malaise (marked weakness), as well as the following common causes of acute rash or skin lesions cannot explain the clinical features: herpes zoster, measles, varicella zoster, dengue, zika, herpes simplex, chikungunya, bacterial skin infections, primary or secondary syphilis, molluscum contagiosum, inguinal granuloma, allergic reactions, and any other common causes associated with localized papules or blisters. The patients fulfilling these criteria are considered suspected cases, which are then further classified as probable or confirmed cases based on the results of epidemiology and laboratory tests.

It is important to differentiate MPX from smallpox, cowpox, chickenpox, herpes zoster, eczema, syphilis, herpes simplex, yaws, scabies, measles, rickettsia pox, bacterial skin infections, and drug-associated rashes [40]. The identification of MPX and these diseases is based on whether there is a history of living in MPX-epidemic areas, coming in contact with infected animals or MPX patients, showing typical symptoms, the time of rash onset, and self-limiting disease course. However, ultimately, laboratory test results are essential for confirmed diagnosis and differentiation. Enlarged lymph nodes are a distinctive feature of MPX, which helps distinguish it from similar symptom-presenting diseases such as chickenpox, measles, and smallpox [25].

Notably, there are always some differences between the clinical summaries of cases when compared between outbreaks since the discovery of MPX. For instance, WHO reported several cases of MPX in nonendemic countries and regions in 2022 that did not have the typical clinical features of fever and swollen lymph nodes followed by a centrifugal rash; rather, they exhibited several atypical signs and symptoms, including few or even only a single skin lesion, the lack of skin lesions presenting as anal pain and bleeding, lesions contained in the genital or perineal/perianal area, skin lesions preceding fever, discomfort, and other systemic symptoms (no prodromal phase). It has been suggested that this single genital lesion, oral, or anal mucosal ulcer is a new manifestation of MPX that should be added to the clinical description of this disease to help diagnose suspected and probable cases in a timely manner [41,42].

### Treatment

#### Supportive treatment

The treatment approach for MPX infection is mainly symptomatic and supportive to alleviate the symptoms, manage complications, and prevent long-term sequelae [43], including replenishment of nutrition and water; pain control with antipyretics/analgesics; keeping the oral cavity, eyes, nose, and skin clean; early identification of secondary infections; and treat with appropriate antibiotics promptly. MPX is a self-limiting disease with a good prognosis in most cases. Severe cases are, however, common in young children and immunocompromised individuals.

#### Antiviral therapy

Anti-smallpox virus drugs can play the role of anti-MPX. WHO recommends that antiviral drugs be used to treat MPX patients presenting with severe symptoms or those who are at high risk for progression to the severe stage, or MPX patients presenting with infections of the eye, mouth, and other specific sites (for instance, genitals or anus), usually using tecovirimat, cidofovir, or brincidofovir (**Table 4**). It is unknown whether people with severe MPX infection will benefit from either antiviral, randomized clinical trials are needed to assess the efficacy of these drugs, and such trials are being implemented by WHO and several countries, especially with tecovirimat [44].

**Table 4:** Treatment and prevention of MPX.

Name	Mechanism of action	Administration	Indications	Contraindications	Common side effects	Serious adverse events	Application in MPX
**Antiviral medicine**							
Tecovirimat (TPOXX, ST-246)	Tecovirimat targets and inhibits the activity of the orthopoxvirus VP37 protein and blocks its interaction with cellular Rab9 GTPase and TIP47, which prevents the formation of egress competent enveloped virions necessary for cell to cell and long-range dissemination of virus	PO, IV	·Treatment of human smallpox disease in adults and pediatric patients weighing at least 13 kg (FDA, 2018) ·Monkeypox (European Medical Association, 2022) ·Healthcare providers can provide tecovirimat to MPX patients under IND protocol(USCDC, 2022)	Patients with severe renal impairment (defined as creatinine clearance below 30 mL/min)	Headache, nausea, abdominal pain, vomiting, and administration site reaction	None	·Can be considered for prophylactic use in an exposed person with severe immunodeficiency in T-cell function for which smallpox or MPX vaccination following exposure to MPX virus is contraindicated.
Cidofovir(Vistide)	Cidofovir suppresses cytomegalovirus (CMV) replication by selective inhibition of viral DNA synthesis. Biochemical data support selective inhibition of CMV DNA polymerase by cidofovir diphosphate, the active intracellular metabolism of cidofovir	IV	·Treatment of CMV retinitis in patients with Acquired Immuredeficiency Syndrome(FDA, 1996)	·Patients with serum creatinine >1.5 mg/dL, a calculated creatinine clearance ≤55 mL/min, or a urine protein ≥100 mg/dL (equivalent to ≥2+ proteinuria). ·Patients receiving agents with nephrotoxic potential	Proteinuria, neutropenia, fever, neutropenia, acidosis	Renal impairment	·It is unknown whether a person with severe MPX infection will benefit from treatment with cidofovir, although its use may be considered in such instances.
Brincidofovir (CMX001, Tembexa)	Brincidofovir is a lipid conjugate of cidofovir. Once inside cells, the lipid ester linkage of brincidofovir is cleaved to liberate cidofovir, which is then phosphorylated to produce cidofovir diphosphate, which selectively inhibits orthopoxvirus DNA polymerase-mediated viral DNA synthesis.	PO	·Treatment of human smallpox disease in adult and pediatric patients, including neonates.(FDA, 2021) ·Brincidofovir is made available from the Strategic National Stockpile (SNS) for treatment of MPX to clinicians who request and obtain an FDA-authorized single-patient emergency use IND (e-IND).(USCDC, 2022)	None	Diarrhea, nausea, vomiting, and abdominal pain	None	·Brincidofovir can be considered for patients having severe disease or are at high risk for progression to severe disease, or patients with poor therapeutic effect of tecovirimat or otherwise ineligible for tecovirimat
**Blood product**							
Vaccinia Immune Globulin Intravenous (VIGIV)	VIGIV provides passive immunity for individuals with complications to vaccinia virus vaccination.	IV	·Treatment of complications due to vaccinia vaccination(FDA, 2005) ·CDC holds an expanded access IND protocol that allows the use of stockpiled VIGIV for the treatment of orthopoxviruses (including MPX) in an outbreak.(USCDC, 2022)	·Isolated vaccinia keratitis. ·Individuals with a history of anaphylaxis or prior severe systemic reaction associated with the parenteral administration of this or other human immune globulin preparations. ·IgA-deficient patients with antibodies against IgA and a history of IgA hypersensitivity.	Headache, nausea, rigors, and dizziness	Severe vaccinia infection	·Healthcare providers may consider its use in severe cases where the development of a robust antibody response may be impaired. ·Can be considered for prophylactic use in an exposed person with severe immunodeficiency in T-cell function for which smallpox or MPX vaccination following exposure to MPX virus is contraindicated.
**Vaccines**							
ACAM 2000	A live attenuated vaccinia virus vaccine derived from a plaque-purified clone can induce an effective immune response.	Percutaneous, using a bifurcated needle, single dose	·Active immunization against smallpox disease for persons determined to be at high risk for smallpox infection (FDA, 2007) ·Available for use against MPX under an EA-IND protocol (USCDC, 2022)	·History of a severe allergic reaction after a previous dose of ACAM2000 ·Three or more major cardiac risk factors ·Eye disease treated with topical steroids ·Congenital or acquired immune deficiency disorders, including those taking immunosuppressive medications and people living with HIV ·Atopic dermatitis/eczema and people with a history of atopic dermatitis/eczema or other acute or exfoliative skin conditions ·Pregnancy or breastfeeding ·Infants age <12 months	Inoculation site signs and symptoms, lymphadenitis, and constitutional symptoms, such as malaise, fatigue, fever, myalgia, and headache	Myocarditis and/or pericarditis, progressive vaccinia in immunocompromised persons, eczema vaccinatum in persons with skin disorders, auto- and accidental inoculation, generalized vaccinia, urticaria, erythema multiforme major (including Stevens-Johnson syndrome) and fetal vaccinia in pregnant women	· Preexposure indications:prophylaxis (PrEP) is recommended for health workers at risk, clinical laboratory staff performing diagnostic testing, laboratory personnel working with orthopoxviruses, and others at risk under national policy · Postexposure indications:ideally within 4 days of the first exposure · ACAM2000 will be made available for individuals who decide in consultation with their healthcare provider that the potential benefits of vaccination outweigh any potential risks from ACAM2000 adverse events
JYNNEOS (MVA, Imamune, Imvanex)	An attenuated, live, nonreplicating smallpox and MPX vaccine that elicits humoral and cellular immune responses to orthopoxviruses.	Two subcutaneous or intradermal doses, 28 days apart	·Prevention of smallpox and MPX disease in adults 18 years of age and older determined to be at high risk for smallpox or MPX infection.(FDA, 2019) ·People aged <18 years with the above conditions under an Emergency Use Authorization.(USCDC, 2022) ·IMVANEX for MPX prevention(EMA, 2022) ·IMAMUNE for immunization against MPX and Orthopox virus(PHAC, 2020)	·History of a severe allergic reaction (for instance, anaphylaxis) after a previous dose of JYNNEOS	·Injection site reactions(pain, redness, swelling, induration, and itching) ·Systemic adverse reactions(muscle pain, headache, fatigue, nausea, and chills)	Serious adverse events reported in clinical trials and a causal relationship to JYNNEOS could not be excluded: Crohn’s disease, sarcoidosis, extraocular muscle paresis and throat tightness(all nonfatal)	·Pre- and postexposure indications are the same as above ·Safer than ACAM2000 and can be vaccinated regardless of pregnancy, breastfeeding, or weakened immune system when necessary
LC16m8	A live, minimally replicating attenuated smallpox vaccine	Percutaneous, using a bifurcated needle, single dose	·Active immunization against smallpox (Japanese authorities, 1975) ·Active immunization against MPX(Japanese authorities, 2022)	·History of a severe allergic reaction after a previous dose of LC16m8	Lymphadenopathy, fever, and injection site reactions (erythema and swelling)	None.	·Pre- and postexposure indications are the same as above ·Safer than ACAM2000 and can be vaccinated in those with contraindications for replicating vaccines, immune deficiencies, or atopic dermatitis ·Preferred for pregnant women if modified vaccinia virus Ankara-Bavarian Nordic not available ·Licensed in Japan for use in children

#### Tecovirimat

Tecovirimat (TPOXX, ST-246), a smallpox virus treatment drug approved by the FDA in 2018, possesses strong inhibitory activity against orthopox viruses such as the smallpox virus, MPX, and cowpox virus. In 2022, tecovirimat was approved for the treatment of MPX in Europe [45]. The US CDC holds expanded access to the Investigational New Drug (IND) protocol, which allows the use of tecovirimat to treat MPX during an outbreak. Tecovirin is available in both oral capsule and intravenous formulations; the oral form is to be used twice daily for 14 days.

Tecovirimat is highly efficient on the MPX lineage responsible for the 2022 global outbreak in vitro [46] and which has been demonstrated to be effective in the animal study [47]. On the other hand, the results of a recent observational study involving a very small number of patients suggest that tecovirimat may have effectively shortened the illness time [48]. In addition, data collected from 25 cases with diagnosed MPX infection who had completed a course of therapy with tecovirimat suggested the good tolerance of this antiviral drug in all cases, albeit with minor side effects [49].

#### Cidofovir

Cidofovir acts by inhibiting viral DNA polymerase, and it is approved by the FDA for the treatment of cytomegalovirus. A study on a nonhuman primate model showed a significant reduction in the extent of cutaneous MPX lesions and mortality with cidofovir treatment initiated 24 h after a lethal intratracheal MPX infection [50]. Cidofovir is recommended only for use in critically ill patients since its limitations including lack of oral bioavailability [51] reported nephrotoxicity [52].

#### Brincidofovir

Brincidofovir—a prodrug of cidofovir—was licensed by the FDA for treating smallpox in 2021, with less renal toxicity [53]. Brincidofovir has demonstrated good antiviral ability in an MPX animal model [54], suggesting that early treatment with this drug in MPX patients is likely to have better outcomes, albeit there is no evidence supporting this drug’s effectiveness in the treatment of human MPX. A retrospective analysis in the UK indicated that tecovirimat was more effective than brincidofovir in a limited number of human MPX cases [48]. Brincidofovir has been made available from the Strategic National Stockpile (SNS) to clinicians who requested and obtained an FDA-authorized single-patient emergency use IND (e-IND) for the treatment of MPX. Given its approval for the treatment of pediatric patients, brincidofovir will complement tecovirimat to ensure a robust supply of the therapeutic drug.

Brincidofovir is administered as an oral tablet or a suspension and may cause elevated transaminases and gastrointestinal adverse effects, including diarrhea, nausea, vomiting, and abdominal pain. This effect is usually reversible and does not require discontinuation of the drugs, albeit 3 patients treated with brincidofovir developed elevated liver enzyme levels, resulting in the cessation of therapy in the UK [48]. The liver function tests are therefore recommended before and during treatment with this drug.

#### Vaccinia Immune Globulin Intravenous (VIGIV)

VIGIV is a specific antibody produced by individuals who have been vaccinated against smallpox [55], and it is approved by the FDA for the treatment of complications arising from VACV vaccination, including progressive vaccinia, severe generalized vaccinia, eczema vaccinatum, and vaccinia infections in individuals with skin conditions and aberrant infections induced by VACV (except in cases of isolated keratitis). There are no data on the effectiveness of its use in the treatment of MPX infection, and it remains unclear whether severe cases of MPX would benefit from it. For individuals exposed to MPX and with a T-cell functional immune deficiency, when the smallpox vaccine cannot be inoculated, VIGIV should be used for prevention.

#### Other antiviral therapies

In addition, some DNA polymerase inhibitors and nucleoside analogs also demonstrated inhibitory activity against OPV in vitro [56,57]. Nioch-14, a synthetic analogue of tecovirimat, is considered a potential anti-MPX drug, offering the advantage of ease of production [58].

### Prevention

#### General prevention

In response to the current MPX epidemic, there is a need to effectively prevent and control the spread of MPX through active control measures, including prompt identification and isolation of cases, nonpharmaceutical shielding methods in the known transmission routes, vaccination of close contacts (ring vaccination) [59]. Public health organizations should promote and raise awareness about MPX, especially training health workers to rapidly identify, isolate, and manage patients with MPX.

#### Prevention with vaccines

Until date, no specific vaccine has been established for MPX. In fact, smallpox vaccines were used in the current outbreak of MPX considering that it offers immunological cross-protection among OPVs [60]. It has also been proven that the first-generation live vaccinia vaccines can provide approximately 85% protection against MPX infection [61]. There are currently 3 classes of licensed smallpox vaccines (JYNNEOS, LC16, and ACAM2000) (**Table 4**), JYNNEOS was the main vaccine in use during the current outbreak, which is approved for the prevention of smallpox and MPX.

Individuals at a higher risk for exposure to MPX or those who have been exposed to MPX may be offered vaccination to prevent MPX disease; these measures are called preexposure and postexposure prophylaxis, respectively, and are used safely and effectively by the public health departments in the USA, the UK, and Singapore [62–64]. Based on the recommendation of the USCDC, vaccination within 4 days of exposure can prevent the onset of the disease and may prevent MPX [65], while vaccination 4 to 14 days after exposure may reduce the disease severity if the infection occurred [66].

WHO does not recommend mass MPX vaccination based on current risk and benefit assessment. The smallpox vaccine is not available privately or commercially and remains the property of the government. In all cases, clinicians need to pay attention to whom might be eligible for vaccination, such that consultation can then occur with national health authorities in regard to releasing vaccines from national stockpiles in the US or Canada. Currently, ACAM2000, Imvamune, and LC16 are not approved for use in the general population [1]. Therefore, there is still a need to develop an effective and safe next-generation MPX-specific vaccine.

### ACAM2000

The second-generation vaccine ACAM2000 is a live, attenuated, replicating vaccine that has been proven effective against MPX in animal models [67–69]. However, this vaccine can induce serious adverse effects in vaccinated individuals due to its potential for replication, including myocarditis and eczema vaccinatum. Therefore, as a preventive step, ACAM2000 has been contraindicated in individuals with severe immunodeficiency [1].

### JYNNEOS

JYNNEOS is a third-generation smallpox vaccine and a replication-deficient attenuated vaccine called modified vaccine Ankara (MVA, JYNNEOS in the US; IMAMUNE in Canada; IMVANEX in the European Union). It is safer than ACAM2000 and protects animals from MPX [69–72]. In addition, it confers 100% protection against MPX [68] and provides long-term protective immunity [73] against lethal MPX challenge, as demonstrated in macaque challenge trials. JYNNEOS is the first and only vaccine to receive FDA approval for MPX prophylaxis. Some data on the performance of the JYNNEOS vaccine in the current outbreak are gradually becoming available. Across 32 USA jurisdictions, men aged 18 to 49 years were deemed eligible for JYNNEOS vaccination. The MPX incidence was reportedly 14 times higher among nonvaccinated men when compared with that among those who had received a first vaccine dose ≥14 days earlier.

Unlike ACAM2000, JYNNEOS can be used in people with contraindications for replicating vaccines, such as people with immunosuppression, immune deficiencies, or atopic dermatitis, making it preferable for pregnant or breastfeeding women [74].

### LC16m8

LC16m8 is another third-generation vaccine. It is a live, attenuated, low-replicating smallpox vaccine, which was licensed for smallpox prevention in Japan in 1975 and approved for smallpox prevention by the USFDA through the EA-IND protocol in 2014. A study on a nonhuman primate model demonstrated that LC16m8 exhibited long-lasting protection against MPX due to the IgG antibody response and the reduction in viremia [75]. Notably, research on another nonhuman primate model validated that LC16m8 is a safer and more effective alternative to ACAM2000 and Dryvax (a first-generation vaccine) for immunocompromised individuals [76]. Furthermore, LC16m8 has been attributed to a good safety profile with low frequencies of minor adverse events and no serious adverse events [77], making it safe for use in infants, children, and adults, including pregnant and lactating women.

## Conclusions

Human MPX poses a major threat to human health. There are many possible reasons for this unusual outbreak of MPX, including a decline in the proportion of people vaccinated against smallpox, increasingly frequent international travel, and a mutation in the adaptation of the MPX to the human. This MPX outbreak is larger and more widespread than in the past, and the full impact of the disease on a broader population (elderly, children, immunocompromised patients, pregnant women) remains uncertain.

There is also a major concern that MPX may establish animal hosts outside of West or Central Africa. This reservoir of virus could occur in rodents, groundhogs, or the exotic small pet trade. If such animal hosts are established, this would mean that the disease could not be eliminated and would add novel and ongoing risks to the population. Another concern is that the potential evolution of the MPX genome could have one or more of the following effects: increased transmission, enhanced virulence, or reduced antiviral efficacy by altering the gene sequences of proteins inhibited and targeted by antiviral drugs such as VP37 protein and tecovirrimat.

Given the serious threat of MPX, a national framework for teaching, testing, follow-up, and treatment should be widely established: Firstly, raising awareness and educating public, especially at-risk groups, to prevent infection and reduce transmission and spread, frontline healthcare workers around the world should be trained to rapidly identify, isolate, and manage patients with MPX. Secondly, countries need to reassess their preparedness for outbreaks such as MPX and build their own national strategic stockpiles. Resources for training, prevention, diagnosis, surveillance, and treatment cannot be intermittent. Thirdly, in terms of establishing a more global and effective laboratory network, strengthening international specimen referral capacity and developing rapid and sensitive detection tests to improve diagnosis and, thus, prevention. Finally, further research is needed to elucidate the effectiveness and safety of current antiviral treatments and vaccines. Additional research is also needed to develop new diagnostic methods.

**Key Learning Points**

- The monkeypox (MPX) epidemic in 2022 poses a major threat to human health with over 80,000 confirmed cases reported in over 100 countries from May to December 2022.
- The crucial host cell signaling pathways involved in MPX invasion including MAPK/ERK, PAK-1, and PI3K/Akt may provide potential targets and information for the treatment, prevention, and control of the epidemic.
- Combination of multiple methods focusing on “clinical symptoms, epidemiology, and laboratory tests” is indispensable for an accurate diagnosis of MPX, while the laboratory testing techniques are essential to confirm the diagnosis of MPX.
- The treatment approach for MPX infection is mainly symptomatic and supportive; anti-smallpox virus drugs can be used to treat severe cases.
- Timely identification and isolation of MPX cases, cutting off the transmission route, and vaccination of close contacts (ring vaccination) are effective measures for prevention and control of MPX transmission.

**Top Five Papers**

1. Farahat RA, Sah R, El-Sakka AA, Benmelouka AY, Kundu M, Labieb F, et al. Human monkeypox disease (MPX). Infez Med. 2022;30(3):372–391.
2. Mitja O, Ogoina D, Titanji BK, Galvan C, Muyembe JJ, Marks M, et al. Monkeypox. Lancet. 2022.
3. Beer EM, Rao VB. A systematic review of the epidemiology of human monkeypox outbreaks and implications for outbreak strategy. PLoS Negl Trop Dis. 2019;13(10):e0007791.
4. Huang Y, Mu L, Wang W. Monkeypox: epidemiology, pathogenesis, treatment and prevention. Signal Transduct Target Ther. 2022;7(1):373.
5. Petersen E, Abubakar I, Ihekweazu C, Heymann D, Ntoumi F, Blumberg L, et al. Monkeypox—Enhancing public health preparedness for an emerging lethal human zoonotic epidemic threat in the wake of the smallpox post-eradication era. Int J Infect Dis. 2019;78:78–84.
